# Free, Conjugated, and Bound Phenolics in Peel and Pulp from Four Wampee Varieties: Relationship between Phenolic Composition and Bio-Activities by Multivariate Analysis

**DOI:** 10.3390/antiox11091831

**Published:** 2022-09-16

**Authors:** Xue Lin, Yousheng Shi, Pan Wen, Xiaoping Hu, Lu Wang

**Affiliations:** 1 School of Food Science and Engineering, Hainan University, Haikou 570228, China; 2 Key Laboratory of Food Nutrition and Functional Food of Hainan Province, Hainan University, Haikou 570228, China; 3 School of Food Science and Engineering, Hainan Tropical Ocean University, Sanya 572022, China

**Keywords:** wampee varieties, free phenolic, conjugated phenolic, bound phenolic, antioxidant capacities, inhibitory activity against *α*-glucosidase, multivariate analysis

## Abstract

Free, conjugated, and bound phenolic fractions of peel and pulp in four wampee varieties from South China were analyzed for their contents, composition, antioxidant capacities, and inhibitory activities against *α*-glucosidase. We found that there were significant differences in phenolic/flavonoid contents among diverse varieties and different parts (peel and pulp), and the contents were highest in the peel’s bound form. The results of UHPL-Q-Exactive HF-X and HPLC showed that chlorogenic acid, gentisic acid, and rutin were abundantly distributed over the three phenolic fractions in peel and pulp of all wampee samples, while isoquercitrin was the most abundant in the conjugated form of peel/pulp and myricetin had the richest content in the free form of peel/pulp. Wampee peel had stronger antioxidant capacities of ABTS+, DPPH, ·OH, and FRAP than the pulp, and the bound phenolic fraction of the peel/pulp had much higher antioxidant activities than FP and CP fractions. It is interesting that the same phenolic fraction of the wampee peel displayed roughly close IC_50_ values of *α*-glucosidase inhibition to those from the pulp samples. The relationship between individual phenolic and TPC/TFC/the bio-activities and the similarity among the free, conjugated, and bound phenolic fractions in peel and pulp samples were explored by using Pearson correlation analysis, principal component analysis, and hierarchical cluster analysis. This work provides a systematic and comprehensive comparison of the three phenolic fractions of diverse wampee varieties and different parts, and a rationale for applying phenolics from wampee fruits.

## 1. Introduction

*Clausena lansium* (Lour.) Skeels (wampee) belongs to the genus *Clausena* in the family *Rutaceae*, originating from Southern China [1], and is mainly cultivated in tropical and subtropical regions of the world, such as Southern China, India, Vietnam, and Thailand [2]. Wampee fruit resembles grapes, with a brown or yellow peel that is usually eaten along with the pulp, and tastes sour and sweet, similar to the flavor of kumquat. The leaf is a spicy substitute for curry leaves in cooking in Sri Lanka and Nigeria [3,4]. Wampee has recently received much attention not only because its fruits and leaves have a special flavor, but also because its root, stem, leaf, fruit, and seed can be used to treat and prevent common cold, cough, asthma, viral hepatitis, gastrointestinal disease, ulcers, bronchitis, and malaria in traditional Chinese and Vietnamese medicine [5,6]. Previous studies about wampee mainly focused on carbazole alkaloids isolated from the roots and stems of wampee and neuroprotective effects of these compounds [3,5,6]; the polyphenol composition, antidiabetic, and lipid-lowering effects of polyphenol extracts of the leaves [7]; the polyphenolic profile and antioxidant activity during the leaf development stages, chemical profiles of the leaves, barks, flower, peels, pulps, and seeds of wampee with liquid chromatography tandem mass spectrometry (LC-MS) untargeted metabolomics [8]; the antioxidant and anticancer activities of wampee peel extracts by using different solvents (ethanol, hexane, ethyl acetate, butanol, and water) [1]; and the isolation and purification of compounds from wampee fruit and antioxidant or amylase inhibitory activity of these compounds [9]. Although the polyphenolic composition in the wampee leaves has been well characterized, information about polyphenolic constituents of free phenolic (FP), conjugated phenolic (CP), and bound phenolic (BP) fractions in the peel and pulp of different wampee varieties is still lacking.

Oxidative stress is an imbalance between the production of reactive oxygen species (ROS) and their elimination in the cells and tissues [10], and it can cause chronic inflammation, which can further mediate several chronic diseases such as insulin resistance, type 2 diabetes mellitus (T2DM), and cardiovascular diseases (CVD) [11]. Polyphenols are natural compounds that are rich in dietary sources such as fruits, vegetables, red wine, tea, coffee, cholates, cereals, and dry legumes [12]. Many researchers have demonstrated that polyphenols exert antioxidant and anti-inflammatory effects both in vitro and in vivo [13,14].

The aim of this study was to systematically investigate polyphenol compositions, antioxidant activities in four different in vitro models, and *α*-glucosidase inhibitory activities of FP, CP, and BP fractions in the peel and pulp of four wampee varieties. The polyphenol constituents of wampee samples were accurately identified and quantified by UHPLC-Q Exactive HF-X and HPLC for the first time. More importantly, the contributions of the individual phenolic compounds to the observed biological activities were explored by Pearson correlation analysis and principal component analysis (PCA). In addition, PCA and hierarchical cluster analysis (HCA) were conducted to classify FP, CP, and BP fractions of different parts (peel and pulp) of four different wampee varieties.

## 2. Materials and Methods

### 2.1. Materials and Reagents

Four wampee fruits samples, W1–W4, were collected in July 2020 from South China, including Guangdong, Guangxi, and Hainan provinces, and the varieties were identified by professionals. Detailed information about W1–W4 is shown in Table 1. All the standards, including o-coumaric acid, gallic acid, gentisic acid, chlorogentic acid, DL-catechin, isoquercitrin, caffeic acid, myricetin, quercein, rutin, kaempferol 7-O-glucoside, quercitrin, and kaempferol, were HPLC-grade (>97%) and purchased from Shanghai Macklin Biochemical Co., Ltd. (Shanghai, China). 4-nitrophenyl *α*-D-glucopyranoside (pNPG), 2,2′-azinobis (3-ethylbenzothiazoline-6-sulfonic acid ammonium salt) (ABTS), 1,1-diphenyl-2-picrylhydrazyl radical (DPPH), 6-hydroxy-2,5,7,8-tetramethylchroman-2-carboxylic acid (Trolox), 2,4,6-tris(2-pyridyl)-s-triazine (TPTZ), and all mobile phase reagents used for liquid chromatography, including ethanol, acetonitrile, formic acid, and 2-propanol (HPLC-grade), were purchased from Shanghai Aladdin Biochemical Technology Co., Ltd. (Shanghai, China). Folin–Ciocalteu phenol reagent was purchased from Sigma-Aldrich Chemical Co., Ltd. (St. Louis, MO, USA). Other reagents applied in the study were of analytical grade and purchased from Sinopharm Chemical Reagent Co., Ltd. (Shanghai, China).

### 2.2. Extraction of FP, CP, and BP Fractions

**Figure 1:**
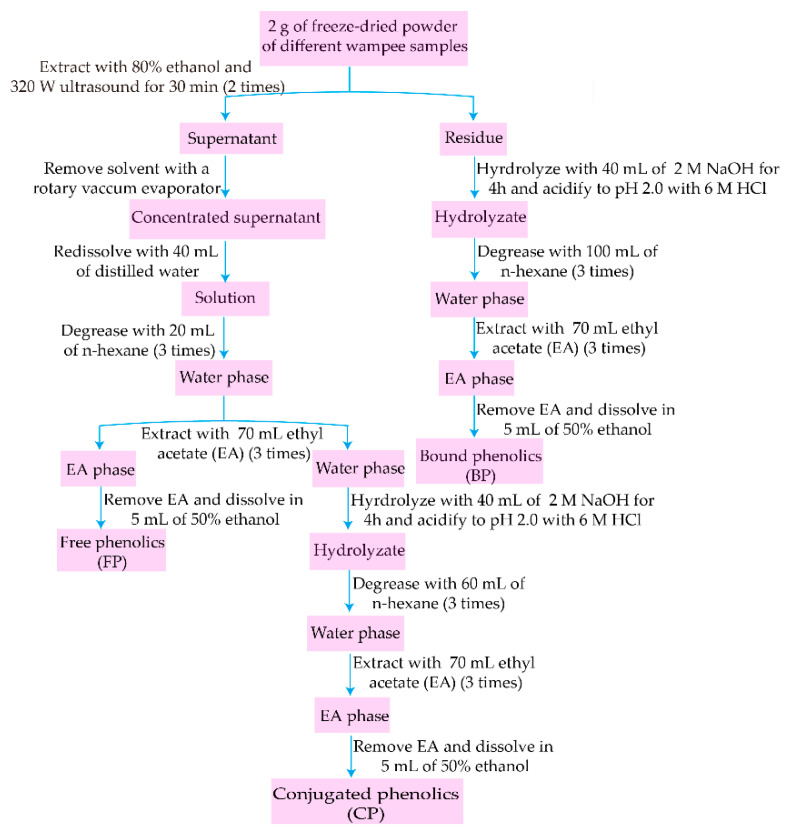
Extraction procedures of free, conjugated, and bound phenolic fractions.

The peel and pulp of W1–W4 were freeze-dried in an LGJ-10 vacuum freeze dryer (Songyuan, Beijing, China). First, dried peel and pulp were ground with a Chinese medicine grinder. Next, they were sieved through a 180 µm mesh, and then the fine peel and pulp powder of W1–W4 were stored at −20 °C until analyses were conducted. Exactions of FP, CP, and BP fractions were performed according to reported methods with some minor modifications [15,16], and the extracting procedure is shown in Figure 1. In brief, freeze-dried powder (2 g) was extracted twice with 25 mL ethanol–water (80:20, *v*/*v*) under ultrasound (320 W) for 30 min. The residue was used for extracting BP, while the supernatant was concentrated, re-dissolved with water (40 mL), degreased with n-hexane (20 mL/time, 3 times), and the water phase was retained to prepare FP and CP. Firstly, the water phase was extracted 3 times with 70 mL/time ethyl acetate (EA). Secondly, EA was removed from EA phase and 50% ethanol (5 mL) was added to obtain FP. Thirdly, the residue and water phase combined from the above two steps were hydrolyzed with 2 M NaOH (40 mL) for 4 h under a nitrogen atmosphere, acidified to pH 2.0 with 6 M HCl, and then degreased 3 times with n-hexane, extracted with EA (70 mL/time, 3 times). Finally, EA was removed and the extractions of CP and BP were prepared by adding 5 mL of 50% ethanol. All samples of peel and pulp from W1–W4 were extracted in three replicates.

### 2.3. Determination of Total Phenolic Content (TPC) and Total Flavonoid Content (TFC)

TPC and TFC of FP, CP, and BP fractions in the peel and pulp of W1–W4 were determined using Folin–Ciocalteu (FC) and aluminum chloride colorimetric methods described by Dou et al. [17]. Gallic acid and rutin were applied as the standards for the determination of TPC and TFC, respectively. TPC and TFC were expressed as mg gallic acid and mg rutin equivalents per gram in dry weight, that is, mg GAE/g DW and mg RE/g DW, respectively. Three biological replicates of all samples obtained as described in Section 2.2 were measured.

### 2.4. Analysis of Polyphenol Composition

#### 2.4.1. Identification of Phenolic Compounds

Phenolic compounds in the FP, CP, and BP fractions from the peel and pulp of W1–W4 were separated by the Vanqusih Horizon UHPLC system (Thermo Scientific, Waltham, MA, USA) equipped with an UHPLC column (Waters ACQUITY UHPLC HSS T3, 100 mm × 2.1 mm i.d., 1.8 µm, Lindon, UT, USA) with eluting solvent A (95% Milli-Q grade water and 5% acetonitrile including 0.1% formic aid) and solvent B (47.5% acetonitrile, 47.5% 2-propanol, and 5% Milli-Q grade water including 0.1% formic aid). The gradient elution procedure was as follows: 0–3.5 min 100–75.5% A; 3.5–5 min 75.5–35% A; 5–5.5 min 35–0% A; 5.5–7.4 min 0% A; 7.4–7.6 min 0–48.5% A; 7.6–7.8 min 48.5–100% A; 7.8–10 min 100% A. Flow rate was 0.4 mL/min, column temperature was set at 40 °C, and injection volume was 2 µL. Mass spectrometry (MS) data were obtained in the positive and negative modes from *m*/*z* 70 to 1050 with Q-Exactive HF-X (Thermo Scientific, Waltham, MA, USA) coupled with an electrospray ionization (ESI) source. MS parameters were as follows: a sheath gas flow rate of 50 arb; an aux gas flow rate of 13 arb; a heater temperature of 425 °C; a capillary temperature of 325 °C; a spray voltage of +3500 V or −3500 V; an s-lens RF level of 50; a normalized collision energy of 20 eV, 40 eV, and 60 eV; a full MS resolution of 60,000; and an MS^2^ resolution of 7500. ProgenesisQI (WatersCorporation, Lindon, UT, USA) was used to obtain retention times, *m*/*z*, and peak intensities of mass spectra. In addition, phenolic compounds in the FP, CP, and BP fractions were identified by matching MS and MS^2^ information with some public databases and literature works. Three biological replicates of all samples obtained as described in Section 2.2 were analyzed.

#### 2.4.2. Quantification of Phenolic Compounds

An Agilent 1200 HPLC system equipped with a column (Agilent Zorbax Eclipse Plus C18, 250 mm × 4.6 mm i.d., 5 µm, Santa Clara, CA, USA) and a diode array detector (Agilent, Santa Clara, CA, USA) was used to quantify the phenolic compounds in the FP, CP, and BP fractions from the peel and pulp of W1–W4. The elution process was performed according to a reported method with minor modifications with eluting solvent A (Milli-Q grade water with 0.1% formic aid) and solvent B (acetonitrile with 0.1% formic aid) [18]. The gradient elution procedure was as follows: 0–6 min 85% A; 6–10 min 85–80% A; 10–20 min 80–75% A; 20–30 min 75–65% A; 30–40 min 65–50% A; 40–55 min 50–20% A; 55–60 min 20–85% A. Other HPLC parameters were as follows: column temperature of 30 °C, injection volume of 10 µL, flow rate of 0.8 mL/min, and detection wavelength of 280 nm. Three biological replicates of all samples obtained as described in Section 2.2 were analyzed.

### 2.5. Evaluation of Antioxidant Activities in Four In Vitro Models

ABTS+, DPPH, and OH radical scavenging activities and ferric-reducing antioxidant power (FRAP) were used to evaluate the antioxidant activities of the FP, CP, and BP fractions from the peel and pulp of W1–W4. The antioxidant activity assays in the four models were conducted according to previous studies [19,20]. Trolox was a positive control in the first three models, and the results were all expressed as µmol Trolox equivalents per gram in dry weight (µmol Trolox/g DW). In the FRAP model, FeSO_4_ was used to establish a standard curve, and the antioxidant activity was expressed as µM Fe(II)SE/g DW. Three biological replicates of all samples obtained as described in Section 2.2 were measured.

### 2.6. Determination of α-Glucosidase Inhibitory Activity

The *α*-glucosidase inhibitory activities of FP, CP, and BP fractions from the peel and pulp of W1–W4 were determined by the method described by Cai et al. [21]. Firstly, 1 U/mL *α*-glucosidase solution was prepared with 0.1 M phosphate buffer solution (PBS, pH 6.8). Secondly, 100 µL of the enzyme solution was mixed with 50 µL of FP, CP, and BP extracts and incubated at 37 °C for 10 min. Thirdly, 100 µL of 5 mM pNPG solution was added and incubated at 37 °C for 20 min. Finally, the absorbance of the reaction mixture was detected at 405 nm in 15 min.

In the model, acarbose and PBS (pH 6.8) were the positive and negative controls, respectively. Inhibitory activity against *α*-glucosidase was expressed as IC_50_ (µg/mL), referring to the amount of the FP, CP, and BP fractions required to inhibit 50% of *α*-glucosidase activity. Three biological replicates of all samples obtained as described in Section 2.2 were analyzed.

### 2.7. Statistical Analysis

All the detections were conducted in triplicate, and the data were expressed as mean ± standard deviation (SD). One-way analysis of variance (ANOVA) and Pearson correlation analysis were performed using SPSS statistics 23 (IBM, Chicago, IL, USA), and graphs were generated using GraphPad Prism 8 (GraphPad Software, San Diego, CA, USA) and R 3.6.3 (ggplot2 package). SIMCA 14.1 (MKSUmetrics, SE) and R 3.6.3 (ggplot2 and ggdendro packages) were used to perform principal component analysis (PCA) and hierarchical cluster analysis (HCA) and visualize related graphs.

## 3. Results and Discussion

### 3.1. Total Phenolic Contents (TPC) and Total Flavonoid Contents (TFC)

**Figure 2:**
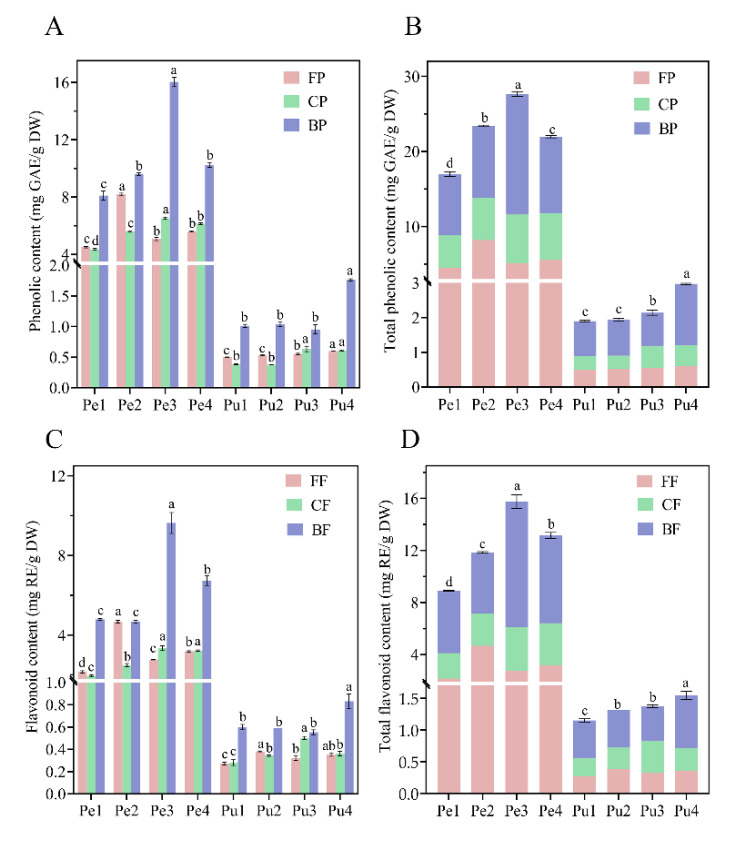
Free, conjugated, and bound phenolic contents (**A**); total phenolic contents (**B**); the free, conjugated, and bound flavonoid contents (**C**); total flavonoid contents (**D**) in the peel and pulp of different wampee samples from South China. Different letters (a–d) indicate significant differences among free, conjugated, and bound phenolic fractions of wampee peel/pulp samples. Pe, wampee peel; Pu, wampee pulp; FP, free phenolic; CP, conjugated phenolic; BP, bound phenolic; FF, free flavonoid; CF, conjugated flavonoid; BF, bound flavonoid.

There were significant differences among the contents of free phenolic (FP), conjugated phenolic (CP), bound phenolic (BP), free flavonoid (FF), conjugated flavonoid (CF), and bound flavonoid (BF) in the peel and pulp of different wampee samples, as shown in Figure 2. TPC and TFC in the four wampee peel samples were in the ranges of 16.99–27.64 mg GAE/g DW and 8.90–15.75 mg RE/g DW, respectively; TPC and TFC in the four pulp samples were 1.90–2.97 mg GAE/g DW and 1.15–1.55 mg RE/g DW, respectively. It was found that the contents of FP and CP in peel or pulp of the four wampee samples resembled one another, and similar contents of FF and CF were also observed in the same peel or pulp samples. Moreover, the BP or BF was dominant in the TPC or TFC, accounting for approximately 50–65% of the total contents. BP/BF contents and TPC/TFC in the peel of Jixin wampee or in the pulp of Heipi wampee were significantly higher than in other genotype samples.

Prasad et al. [1] confirmed that the phenolic contents of hexane fraction, ethyl acetate fraction, butanol fraction, and water fraction of wampee peel were 7.9 µg GAE/g DW, 330 µg GAE/g DW, 30.3 µg GAE/g DW, and 54 µg GAE/g DW, respectively. The total FP content from the above four fractions was 4.22 mg GAE/g DW, lower than those of the samples in the current study (4.52–8.20 mg GAE/g FW). Chang et al. [22] reported that the average FP and FF of peel and pulp mixture from five sour wampees were 5.22 mg GAE/g FW and 4.81 mg CE/g FW, and they were 6 times and 4 times higher than those of five sweet wampee varieties, respectively. In this research, Jixin (W3) is a sour wampee and Seedless (W1) is a sweet wampee, and we found 1.2- to 2.2-fold differences in the contents of FP, CP, BP, FF, CF, and BF of the peel or pulp from the four wampee samples. This discrepancy may be due to the diverse genotypes and growth environment.

Previous studies have mainly focused on the soluble FP of plants; however, CP and BP have been less studied. CP is soluble and can be bound to soluble oligo-saccharides or peptides by hydrophobic, covalent ester, and ether bonds, while BP is insoluble and can be covalently bound to cellulose, hemicellulose, and lignin [16,23,24,25]. Many studies demonstrate that significant amounts of CP and BP are released by colonic fermentation, and the released phenolics may play a significant role in gut health [23,26,27]. In this work, the contents of CP and BP from peel/pulp of the wampee samples accounted for 75–80% of the TPC; thus, the total phenolic content and biological activities have been seriously underestimated. The two phenolic fractions may be the predominant contributors to the delivery of antioxidants to the colon.

### 3.2. Identification of Phenolic Compounds

Phenolic compositions of wampee peel and pulp were analyzed by UHPLC coupled with Q-Exactive HF-X, and the total ionic chromatograms are shown in Figure S1. By comparing parent ions, MS^2^ fragment ions, and retention times with public databases and standards, a total of 19 polyphenols were identified, as shown in Table 2. Some of them belonged to flavonoids, including epigallocatechin, (+/−)-taxifolin, DL-catechin, isoquercitrin, (+)-epicatechin, myricetin, quercetin, rutin, fisetin, populnin (i.e., kaempferol 7-O-glucoside), quercitrin, kaempferol, and (+/−)-naringenin. The others were phenolic acids, including o-coumaric acid, gallic acid, gentisic acid, chlorogenic acid, m-salicylic acid, and caffeic acid.

Compounds **1** and **5** were easily identified as o-coumaric acid and chlorogenic acid with molecular ions at *m*/*z* 165.0549 [M+H]^+^ and 353.0883 [M-H]^−^, respectively, as well as MS^2^ fragment ions at *m*/*z* 123.0444 and 191.0543, based on the MS information from public database (MassBank of North America). Compounds **2**, **3**, and **10** were identified as gallic acid, gentisic acid, and caffeic acid with parent ions at *m*/*z* 169.0136 [M-H]^−^, *m/z* 153.0185 [M-H]^−^, and *m*/*z* 179.0344 [M-H]^−^, respectively. Moreover, all the three phenolic acids lost CO_2_, and created fragment ions at *m*/*z* 125.0234 [(M-H)-CO_2_]^−^, m/*z* 109.0283 [(M-H)-CO_2_]^−^, and *m/z* 135.0441 [(M-H)-CO_2_]^−^, respectively. These phenolic acids had similar fragment patterns to the ones reported by Zhu et al. [18], Arruda et al. [28], and Wang et al. [29].

Compounds **4** and **6** exhibited precursor ions at *m*/*z* 305.0669 [M-H]^−^, 303.0514 [M-H]^−^, and fragment ions at *m*/*z* 125.0234, 137.0229 and 125.0233, 258.0435. Therefore, they were easily identified as epigallocatechin and taxifolin by comparing the fragment patterns with the data from MassBank Europe and MassBank of North America, respectively. Compound **7** was characterized as m-salicylic acid by the molecular ion at *m*/*z* 139.0392 [M+H]^+^, and MS/MS fragment ions at *m*/*z* 121.0284 [(M+H)-H_2_O]^+^ and *m*/*z* 95.0493 [(M+H)-CO_2_]^+^, due to the loss of H_2_O and CO_2_. Compound **8** was identified as DL-catechin according to the similar parent ion at *m*/*z* 289.0721 [M-H]^−^ and fragment ions at 245.0468, 205.0492, and 109.0286 described by Li et al. [30]. Based on the molecular ion at *m*/*z* 335.0777 [M+FA-H]^−^, which was equal to MW 290, compound **11** was tentatively identified as (+)-epicatechin. Compound **12** exhibited a parent ion at *m*/*z* 319.0453 [M+H]^+^ as well as fragment ions at *m*/*z* 245.0468 [(M+H)-C_3_H_6_O_2_]^+^, 217.0505 [(M+H)-C_3_H_6_O_2_-CO]^+^, and 153.0187 [(M+H)-C_7_H_2_O_5_]^+^, due to the loss of 74 amu, 102 amu, and 166 amu. According to the similar fragment pattern, this compound was distinctly identified as myricetin [31]. Similarly, compound **13**, which showed a molecular ion at *m*/*z* 303.0504 [M+H]^+^, with MS^2^ fragment ions at 229.0503 [(M+H)-C_3_H_6_O_2_]^+^, 153.0817 [(M+H)-C_7_H_2_O_4_]^+^, 137.0232 [(M+H)-C_7_H_2_O_5_]^+^, was easily identified as quercetin.

Compounds **15** and **18** were two isomers and tentatively identified as fisetin and kaempferol, according to the same precursor ion at *m*/*z* 287.0555 [M+H]^+^ with MS/MS fragment ions at *m*/*z* 213.0554 [(M+H)-C_3_H_6_O_2_]+, 137.0242 [(M+H)-C_7_H_2_O_4_]^+^, 121.0289 [(M+H)-C_7_H_2_O_5_]^+^, and MS/MS fragment ions at *m*/*z* 153.0185 [(M+H)-C_7_H_2_O_3_]^+^, 121.0289 [(M+H)-C_7_H_2_O_5_]^+^, respectively. By comparing the fragment patterns (MS/MS ions at *m*/*z* 271.0615 [M-H]^−^, 151.0030 and 119.0492) with the data from MassBank Europe, compound **19** was distinctly identified as (+/−)-naringenin.

In view of the molecular ion at *m*/*z* 465.1037 [M+H]^+^ and fragment ions at *m*/*z* 303.0498 [C_15_H_10_O_7_+H]^+^ and 285.0414 [(C_15_H_10_O_7_+H)-H_2_O]^+^, compound **9** was tentatively identified as quercetin 3-O-glucoside. Considering a precursor ion at *m*/*z* 611.1617 [M+H]^+^ and the MS/MS fragment ion at *m*/*z* 303.0501 [C_15_H_10_O_7_+H]^+^, compound **14** was easily identified as rutin. Compounds **16** and **17** were two isomers and identified as kaempferol 7-O-glucoside and quercitrin, because the former was characterized by the parent ion at *m*/*z* 449.1086 [M+H]^+^ and MS/MS fragment ions at 431.0988 [(M+H)-H_2_O]^+^, 287.0529 [C_15_H_10_O_6_+H]^+^. However, the latter exhibited the molecular ion *m*/*z* 447.0938 [M-H]^−^ and MS/MS fragment ions at *m/z* 300.0272 [C_15_H_10_O_7_-2H]^−^, 271.0249, 255.0298, the result being consistent with the fragment pattern of quercitrin from MassBank of North America.

### 3.3. Quantitative Analysis of Phenolic Composition

As shown in Table 3, the contents of gallic acid, DL-catechin, caffeic acid, populnin, quercetin, and kaempferol were much lower than other phenolics in all the peel/pulp from the four wampee varieties. Moreover, BP in the peel of Jixin wampee had significantly higher chlorogenic acid (2611.36 µg/g DW), gentisic acid (1423.05 µg/g DW), rutin (1197.04 µg/g DW), and myricetin (2499.72 µg/g DW) than in other samples, which resulted in higher TPC.

Chlorogenic acid was distributed over FP, CP, and BP of all the samples. In wampee peel, it was mostly concentrated in BP, less in FP, and least in CP, but it was most in BP and with similar content in FP and CP from the wampee pulp. It was found that gentisic acid and rutin almost distributed over FP, CP, and BP in all the peel and pulp samples. They were mostly concentrated in CP, less in BP, and least in FP. Isoquercitrin mainly existed in CP, and had similar content in FP and BP from almost all the peel and pulp samples. Quercitrin was much more abundant than other phenolics in BP from all the peel and pulp samples. In addition, it was also rich in FP from all peel and pulp samples and in peel CP, but it was not detected in CP of the pulp samples. o-Coumaric acid was rich in FP and CP from almost all peel and pulp samples, but it did not exist in all BP. Myricetin was rich and predominant in FP of wampee peel and pulp and also existed in BP of the peel of Seedless and Jixin wampee. However, it was not detected in CP and BP of the other peel and pulp samples.

Although many studies have measured the content of FP and phytochemical components [22,32,33], information remains lacking about the characterization and contents of FP, CP, and BP of different wampee varieties and different parts. Chang et al. [22] reported that rutin in FP of the mixture of peel and pulp from all ten wampee varieties was richer (>38.41 µg/g FW) than the other seven phytochemical components, including syringin, benzoic acid, 2-methoxycinnamic acid, kaempferol, hesperetin, nobiletin, and tangeretin, and kaempferol with low content was only detected in FP from three sour wampee varieties and less than 2.29 µg/g FW. The contents of rutin and kaempferol in FP were similar to those in the present work. In recent studies, fifteen flavonoids (taxifolin-3-O-rhamnoside and other flavone glycosides) and nine phenolic acids (o-coumaric acid and other chemicals) were identified as different metabolites in the juice extracted from the pulp of sweet, sweet–sour, and sour wampee samples by UPLC-MS/MS-based widely targeted metabolome analysis [32,33]. Most of the 24 components were different from the phenolic composition in this study, which may be due to differences in extraction methods, detection means, and genotypes.

### 3.4. Antioxidant Activities

In this work, three radical scavenging models, ABTS·+, DPPH, and ·OH, as well as another total antioxidant capacity assay (FRAP), were used to measure antioxidant activities of FP, CP, and BP from the peel and pulp of the four wampee varieties, since at least two models should be employed for evaluating in vitro antioxidant capacities [34].

**Figure 3:**
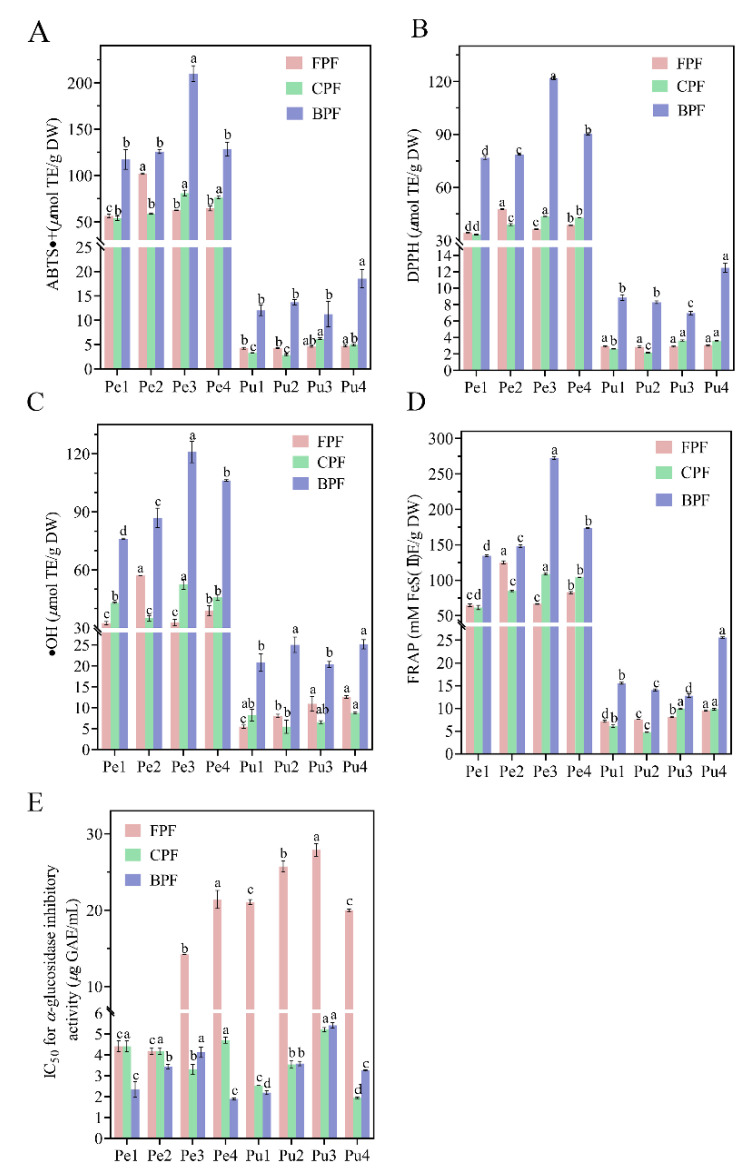
ABTS·+ levels (**A**), DPPH values (**B**), ·OH levels(**C**), FRAP values (**D**), and IC_50_ values of *α*-glucosidase inhibitory activities (**E**) of free, conjugated, and bound phenolic fractions in the peel and pulp of different wampee samples from South China. Different letters (a–d) indicate significant differences among free, conjugated, and bound phenolic fractions of wampee peel/pulp samples. Pe, wampee peel; Pu, wampee pulp; FPF, free phenolic fraction; CPF, conjugated phenolic fraction; BPF, bound phenolic fraction.

As shown in Figure 3A–E, antioxidant activities of FP, CP, and BP from the different wampee varieties and positions varied significantly. Regarding ABTS+ model, the antioxidant levels of FP, CP, and BP in peel of different wampee varieties were 56.32–101.93 µmol TE/g DW, 53.84–80.94 µmol TE/g DW, and 117.32–210.00 µmol TE/g DW, respectively; those of the pulp samples were in the ranges of 4.25–4.73 µmol TE/g DW, 2.85–4.96 µmol TE/g DW, and 11.26–18.56 µmol TE/g DW, respectively. For the DPPH assay, FP, CP, and BP from peel samples yielded antioxidant values of 34.49–47.81 µmol TE/g DW, 33.38–43.68 µmol TE/g DW, and 76.79–121.84 µmol TE/g DW, respectively; those from pulp samples showed the values of 2.85–3.02 µmol TE/g DW, 2.14–3.62 µmol TE/g DW, and 6.97–12.49 µmol TE/g DW, respectively. With regard to the results of ·OH analysis, the antioxidant values of FP, CP, and BP from peel of the four wampee varieties were 32.49–57.03 µmol TE/g DW, 35.05–52.54 µmol TE/g DW, and 76.03–120.94 µmol TE/g DW, respectively. However, those from pulp samples differed were 5.47–12.57 µmol TE/g DW, 5.48–8.76 µmol TE/g DW, and 20.32–25.18 µmol TE/g DW, respectively. In addition, FRAP values of FP, CP, and BP from the peel samples were in the ranges of 64.84–125.23 µmol TE/g DW, 61–108.77 µmol TE/g DW, and 134.60–272.56 µmol TE/g DW, respectively, while those from the pulp samples were in the ranges of 7.18–9.55 µmol TE/g DW, 4.79–9.82 µmol TE/g DW, and 12.78–25.60 µmol TE/g DW, respectively. More importantly, BP of Jixin wampee peel and BP of Heipi wampee pulp exhibited much higher antioxidant capacities in all the four models than other peel or pulp samples. Moreover, antioxidant activities of BP fractions from all peel or pulp samples were much higher than those of CP and FP fractions, and the latter two fractions from each of the samples showed roughly similar antioxidant values. The results indicated that the BP fractions of peel and pulp of the four wampee varieties were the main contributors to the antioxidant capacities.

### 3.5. α-Glucosidase Inhibitory Activity

Diabetes is one of the major causes of disability [35], and type 2 diabetes (T2D) accounts for 90% of all diabetes [36]. The global diabetes prevalence in 2019 was estimated to be 9.3% (463 million people), and this value is expected to rise to 10.2% (578 million) by 2030 and 10.9% (700 million) by 2045 [37]. Therefore, it is obvious that global health spending on diabetes will greatly increase. Until June 2019, metformin and insulin were approved as the only drugs for adult T2D by the U.S. Food and Drug Administration (FDA) [38]. However, metformin can induce gastrointestinal and cutaneous side effects and lactic acidosis [39], and insulin therapy may result in hypoglycemia and weight gain [40,41]. It is therefore urgent to find alternative products from natural compounds without side effects to manage T2D. Many studies have reported that polyphenol intake is related to decreasing the risk of insulin resistance and T2D [13].

It is shown in Figure 3E that FP, CP, and BP from peel and pulp of the four wampee samples exhibited outstanding inhibitory activity against *α*-glucosidase. Due to the lower IC_50_ value with higher *α*-glucosidase inhibition, FP of the pulp from Jixin wampee was the lowest *α*-glucosidase inhibitor (IC_50_ = 27.89 ± 0.83 µg GAE/mL), and BP of the peel from Heipi wampee was the highest *α*-glucosidase inhibitor (IC_50_ = 1.89 ± 0.04 µg GAE/mL) of all the samples. It is worth noting that all the pulp samples from the same phenolic fraction (FP, CP, or BP) displayed roughly similar IC_50_ values to those from the peel samples, but wide variations existed in their phenolic/flavonoid contents and antioxidant activities, as shown in Figure 2 and Figure 3A–D. The abundant flavonoid composition in all the peel and pulp samples may contribute to their strong *α*-glucosidase inhibition. Many studies have confirmed that flavonoid compounds, such as quercetin (IC_50_ = 15.71 µg/mL), myricetin (IC_50_ = 36.17 µg/mL), rutin (IC_50_ = 68.16 µg/mL), and quercitrin (IC_50_ = 113.27 µg/mL), have excellent inhibitory activity against *α*-glucosidase [19,42].

### 3.6. Correlation Analysis and Multivariate Analysis

In this work, Pearson correlation analysis was used to comprehensively explore the relationship between individual phenolic and TPC/TFC/bio-activities (ABTS+, DPPH, ·OH, and FRAP values and IC_50_ value of *α*-glucosidase inhibition) in FP, CP, and BP of peel and pulp from the four wampee varieties.

**Figure 4:**
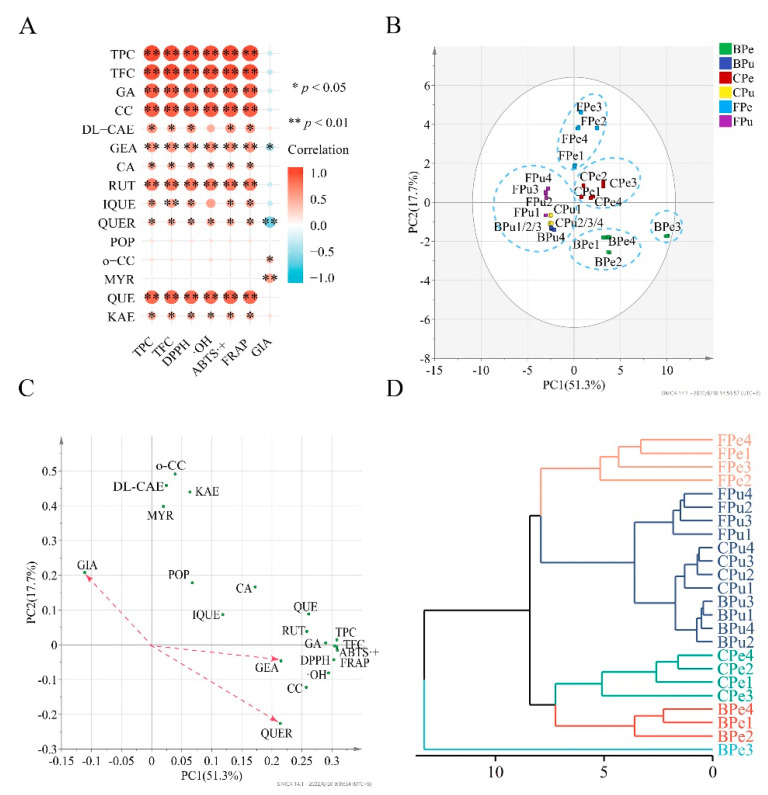
Correlation heatmap (**A**), PCA score plot (**B**), PC loading plot (**C**), and dendrogram (**D**) of the data matrix of the contents of individual phenolic, TPC, TFC, and bio-activities from free, conjugated, and bound phenolic fractions in the peel and pulp of different wampee samples from South China. TPC, total phenolic content; TFC, total flavonoid content; GA, gallic acid; CC, chlorogenic acid; DL-CAE, DL-catechin; GEA, gentisic acid; CA, caffeic acid; RUT, rutin; IQUE, isoquercitrin; QUER, quercitrin; POP, populnin; o-CC, o-coumaric acid; MYR, myricetin; QUE; quercetin; KAE, kaempferol; GIA, *α*-glucosidase inhibition; FPe/FPu, free phenolic fraction of wampee peel/pulp; CPe/CPu, conjugated phenolic fraction of wampee peel/pulp; BPe/BPu, bound phenolic fraction of wampee peel/pulp.

Correlation coefficients of TPC vs. TFC/ABTS+/DPPH/·OH/FRAP/*α*-glucosidase inhibition (GIA) were 0.983 (*p* < 0.01), 0.998 (*p* < 0.01), 0.995 (*p* < 0.01), 0.970 (*p* < 0.01), 0.997 (*p* < 0.01), and −0.291 (*p >* 0.05), respectively. TFC had similar correlation coefficients with ABTS·+ (r = 0.986, *p* < 0.01), DPPH (r = 0.977, *p* < 0.01), ·OH (r = 0.957, *p* < 0.01), FRAP (r = 0.984, *p* < 0.01), and GIA (r = −0.317, *p >* 0.05). It is obvious that the antioxidant activities significantly correlated with TPC and TFC, while GIA did not significantly correlate with them. Moreover, the four antioxidant activities showed significantly positive correlations with the contents of gallic acid, chlorogenic acid, DL-catechin, gentisic acid, caffeic acid, rutin, isoquercitrin, quercitrin, quercetin, and kaempferol and no significant correlation with the contents of populnin, o-coumaric acid, and myricetin. Nevertheless, except for gentisic acid (r = −0.489, *p* < 0.05), quercitrin (r = −0.691, *p* < 0.05), o-coumaric acid (r = 0.441, *p* < 0.05), and myricetin (r = 0.563, *p* < 0.05), other phenolic compounds in the wampee samples were not significantly correlated with GIA (Figure 4A).

Principal component analysis (PCA) and hierarchical cluster analysis (HCA) are widely used for classifying samples into different groups with different algorithms. TPC, TFC, individual phenolic, and bio-activities were used as variables in PCA and HCA, and Euclidean distance was applied in HCA. PC1 and PC2 explained 69% of the total variance in the data; hence, the two principal components can be used for dimensionality reduction. The PCA score plot shows sample–sample similarities; samples can be clustered into a group with high similarity if they are close together, while samples in different groups have high discrepancy. As shown in Figure 4B, peel FP samples, peel CP samples and pulp FP, CP, and BP samples of the four wampee varieties were severally close and separated into three groups. BP samples from the other three varieties’ peels had similar scores and were close together, except for BP of Jixin wampee peel. Therefore, all the samples were classified into five groups. It is interesting that the same result was found in HCA (Figure 4D).

The PCA loading plot displays the relationship among variables. The smaller an acute angle between two variables is, the higher positive correlation they show. However, the larger an obtuse angle between two variables is, the higher negative correlation they possess. A right angle between two variable vectors means they do not correlate. It is shown in Figure 4C that all the acute angles between TPC/TFC and ABTS+/DPPH/OH/FRAP were very small, and each of angles between the contents of populnin/o-coumaric acid/myricetin and ABTS+/DPPH/OH/FRAP was close to 90°. In addition, the obtuse angles between GIA and gentisic acid/quercitrin were the largest two and the acute angles between GIA and o-coumaric acid/myricetin were almost the two smallest. Therefore, the correlations between TPC/TFC/the contents of individual phenolic compounds and ABTS+/DPPH/OH/FRAP/GIA analyzed by PCA were roughly similar to those obtained with Pearson correlation analysis.

## 4. Conclusions

In this work, the contents and phenolic compositions of FP, CP, and BP fractions of peel and pulp from four diverse wampee varieties and their in vitro bio-activities, including antioxidant and *α*-glucosidase inhibitory activities, were reported. The results show that the contents of FP, CP, BP, TPC, and TFC and the antioxidant capacities of all peel samples were much higher than those of all pulp samples. Meanwhile, for peel/pulp from all the wampee varieties, BP/BF was the greatest fraction, accounting for over 50% of TPC/TFC. TPC and TFC in the wampee peel were 16.99–27.64 mg GAE/g DW and 8.90–15.75 mg RE/g DW, respectively, vs. 1.90–2.97 mg GAE/g DW and 1.15–1.55 mg RE/g DW in the wampee pulp. For BP fractions from the wampee peel, the antioxidant values in ABTS+, DPPH, OH, and FRAP models were in the ranges of 117.32–210.00 µmol TE/g DW, 76.79–121.84 µmol TE/g DW, 76.03–120.94 µmol TE/g DW, and 134.60–272.56 µmol TE/g DW, respectively. For BP fractions from the wampee pulp, corresponding values were 11.26–18.56 µmol TE/g DW, 6.79–12.49 µmol TE/g DW, 20.32–25.18 µmol TE/g DW, and 12.78–25.60 µmol TE/g DW, respectively. Chlorogenic acid, gentisic acid, and rutin were abundantly distributed over FP, CP, and BP in wampee peel and pulp. Isoquercitrin was the most abundant in CP of peel/pulp, and myricetin was had richest contents in FP of peel/pulp. Wampee peel showed stronger antioxidant capacities of ABTS+, DPPH, ·OH, and FRAP than the pulp, and BP of the peel/pulp had much higher antioxidant activities than FP and CP fractions. However, the same phenolic fractions (FP, CP, or BP) of the wampee peel displayed roughly similar IC_50_ values of *α*-glucosidase inhibition to those of the pulp samples. Moreover, by using Pearson correlation analysis and PCA, we found that TPC, TFC, and the contents of most of phenolic compounds in the wampee samples were significantly positively correlated with the antioxidant activities, whereas the contents of gentisic acid and quercitrin were significantly negatively correlated with IC_50_ values of *α*-glucosidase inhibition. Except for the BP fraction of Jixin wampee peel, all the other samples were categorized into four classes by PCA and HCA. Overall, BP in peel from all wampee varieties, especially from Jixin wampee, showed high antioxidant activities, and all phenolic fractions (FP, CP, and BP) of the peel/pulp from all wampee samples had excellent inhibitory activities against *α*-glucosidase. Findings from the present study provide a rationale for applying phenolics from wampee fruits.

## Figures and Tables

**Table 1:** Species information of the four wampee samples from South China (n = 6).

Abbre.	Collect Location	Type	Color	Length (mm)	Width (mm)	Single Weight (g)	Photo
W1	Yunfu, Guangdong	Seedless wampee	Bright yellow	31.12 ± 2.78 ac	25.24 ± 2.26 a	10.62 ± 2.72 a	* 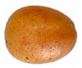 *
W2	Danzhou, Hainan	Wild wampee	Dark yellow	23.61 ± 2.71 c	23.22 ± 1.93 ab	7.15 ± 1.29 b	* 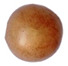 *
W3	Haikou, Hainan	Jixin wampee	Light dark yellow	31.14 ± 2.69 a	21.83 ± 1.81 b	7.63 ± 1.67 b	* 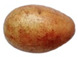 *
W4	Qingzhou, Guangxi	Heipi wampee	Brown	27.48 ± 2.94 b	23.18 ± 2.19 ab	8.38 ± 2.53 ab	* 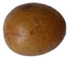 *

Different letters (a–c) indicate significant differences among different wampee varieties (*p* < 0.05).

**Table 2:** Identification of the main phenolic compounds in peel and pulp of the four wampee samples from South China by UHPLC-Q Exactive HF-X.

Peak No.	Retention Time (Min)	Compound Name	Formula	Mw	Molecular Ion (*m*/*z*)	MS/MS (*m*/*z*)	Error	Reference
1	1.38	o-Coumaric acid	C_9_H_8_O_3_	164	165.0549 [M+H]^+^	165.0549, 123.0444,	1.89	Standard, MS/MS
2	1.63	Gallic acid	C_7_H_6_O_5_	170	169.0136 [M-H]^−^	169.0136, 125.0234	−3.73	Standard, MS/MS
3	1.95	Gentisic acid	C_7_H_6_O_4_	154	153.0185 [M-H]^−^	153.0185, 109.0283	−5.14	Standard, MS/MS
4	2.00	Epigallocatechin	C_15_H_14_O_7_	306	305.0669 [M-H]^−^	305.0669, 137.0229, 125.0234	0.91	MS/MS
5	2.18	Chlorogenic acid	C_16_H_18_O_9_	354	353.0883 [M-H]^−^	353.0883, 191.0543	1.42	Standard, MS/MS
6	2.37	(+/−)-Taxifolin	C_15_H_12_O_7_	304	303.0514 [M-H]^−^	303.0514, 285.0435, 125.0233	1.29	MS/MS
7	2.47	m-Salicylic acid	C_7_H_6_O_3_	138	139.0392 [M+H]^+^	139.0392, 121.0284, 95.0493	1.73	MS/MS
8	2.56	DL-Catechin	C_15_H_14_O_6_	290	289.0721 [M-H]^−^	289.0721, 245.0809, 205.0492, 109.0286	1.32	Standard, MS/MS
9	2.96	Isoquercitrin	C_21_H_20_O_12_	464	465.1037 [M+H]^+^	465.1037, 303.0498, 285.0414	1.97	Standard, MS/MS
10	3.03	Caffeic acid	C_9_H_8_O_4_	180	179.0344 [M-H]^-^	179.0344, 135.0441	−2.91	Standard, MS/MS
11	3.29	(+)-Epicatechin	C_15_H_14_O_6_	290	335.0777 [M+FA-H]^-^	335.0777, 179.0342, 135.0441	1.55	MS/MS
12	3.64	Myricetin	C_15_H_10_O_8_	318	319.0453 [M+H]^+^	319.0453, 245.0468, 217.0505, 153.0187	1.88	Standard, MS/MS
13	3.98	Quercetin	C_15_H_10_O_7_	302	303.0504 [M+H]^+^	303.0504, 229.0503, 153.0817, 137.0232	1.76	Standard, MS/MS
14	3.97	Rutin	C_27_H_30_O_16_	610	611.1617 [M+H]^+^	611.1617, 303.0501	1.81	Standard, MS/MS
15	4.41	Fisetin	C_15_H_10_O_6_	286	287.0555 [M+H]^+^	287.0555, 213.0554, 137.0242, 121.0289	1.59	MS/MS
16	4.52	Populnin	C_21_H_20_O_11_	448	449.1086 [M+H]^+^	449.1086, 431.0988, 287.0529	1.77	Standard, MS/MS
17	4.53	Quercitrin	C_21_H_20_O_11_	448	447.0938 [M-H]^+^	447.0938, 300.0272, 271.0249, 255.0298	1.60	Standard, MS/MS
18	5.27	Kaempferol	C_15_H_10_O_6_	286	287.0555 [M+H]^+^	287.0555, 153.0185, 121.0281	1.56	Standard, MS/MS
19	5.64	(+/−)-Naringenin	C_15_H_12_O_5_	272	271.0615 [M-H]^−^	271.0615, 151.0030, 119.0492	1.06	MS/MS

**Table 3:** Phenolic composition in free, conjugated, and bound fractions of peel and pulp of the four wampee samples from South China (*n* = 3).

Phenolic Compounds	Status	Contents (µg/g DW)							
		Pe1	Pe2	Pe3	Pe4	Pu1	Pu2	Pu3	Pu4
Gallic acid (GA)	FP	56.09 ± 1.01 b	87.63 ± 0.83 a	57.81 ± 4.23 b	56.20 ± 1.40 b	25.46 ± 0.05 c	24.05 ± 0.19 cd	22.23 ± 0.05 d	26.20 ± 0.15 c
	CP	45.44 ± 1.65 b	44.48 ± 0.02 b	51.09 ± 0.77 a	41.23 ± 0.70 c	16.70 ± 0.32 e	18.02 ± 0.03 e	20.31 ± 0.04 d	25.11 ± 0.43 c
	BP	102.93 ± 1.86 b	68.96 ± 1.97 d	215.43 ± 1.80 a	97.68 ± 2.97 c	ND	ND	ND	ND
	TP	204.47 ± 0.80 b	201.08 ± 1.12 b	324.32 ± 5.27 a	195.10 ± 3.68 c	42.16 ± 0.27 e	42.07 ± 0.22 e	42.53 ± 0.08 e	51.31 ± 0.28 d
Chlorogenic acid (CC)	FP	316.72 ± 1.14 a	270.71 ± 2.49 b	236.04 ± 11.41 d	255.95 ± 2.83 c	59.15 ± 4.47 e	64.27 ± 4.64 e	58.17 ± 0.83 e	66.28 ± 1.00 e
	CP	234.14 ± 10.93 a	110.65 ± 5.98 d	194.52 ± 7.14 b	165.00 ± 8.19 c	56.84 ± 0.61 f	55.30 ± 0.76 f	53.15 ± 0.20 f	86.97 ± 1.68 e
	BP	626.39 ± 18.87 c	832.93 ± 36.47 b	2611.36 ± 27.81 a	412.30 ± 9.71 d	107.20 ± 0.81 f	126.31 ± 0.67 ef	112.65 ± 0.17 ef	144.65 ± 5.08 e
	TP	1177.25 ± 30.94 b	1214.29 ± 32.98 b	3041.91 ± 46.35 a	833.25 ± 1.31 c	223.19 ± 4.27 e	245.88 ± 3.21 e	223.97 ± 0.46 e	297.90 ± 5.76 d
Gentisic acid (GEA)	FP	251.29 ± 14.39 d	280.42 ± 1.00 c	445.50 ± 5.30 a	357.52 ± 19.08 b	141.54 ± 0.85 g	192.69 ± 10.07 e	119.14 ± 0.35 h	177.92 ± 0.69 f
	CP	1287.14 ± 56.18 c	1412.22 ± 5.30 b	1905.12 ± 228.96 a	1798.06 ± 160.06 a	556.71 ± 25.60 d	285.97 ± 2.48 g	394.77 ± 20.01 f	433.76 ± 1.12 e
	BP	713.60 ± 21.20 d	1068.70 ± 47.70 b	1423.05 ± 19.96 a	921.54 ± 3.03 c	385.18 ± 0.08 e	ND	317.51 ± 0.42 f	252.42 ± 1.06 g
	TP	2252.03 ± 20.59 d	2761.34 ± 52.00 c	3773.67 ± 214.31 a	3077.13 ± 176.11 b	1083.42 ± 24.84 e	478.66 ± 12.55 h	831.41 ± 19.94 g	864.10 ± 0.75 f
Caffeic acid (CA)	FP	1.71 ± 0.01 b	3.77 ± 0.49 a	0.21 ± 0.08 c	3.81 ± 0.11 a	ND	ND	ND	ND
	CP	ND	ND	8.93 ± 0.31 a	ND	ND	ND	ND	ND
	BP	ND	ND	5.43 ± 0.45 a	ND	ND	ND	ND	ND
	TP	1.71 ± 0.01 c	3.77 ± 0.49 b	14.57 ± 0.68 a	3.81 ± 0.11 b	ND	ND	ND	ND
Rutin (RUT)	FP	276.61 ± 8.05 c	813.88 ± 33.73 a	227.27 ± 0.77 d	395.19 ± 11.93 b	30.35 ± 0.78 f	8.37 ± 0.04 h	22.74 ± 0.03 g	36.41 ± 0.06 e
	CP	895.04 ± 6.70 b	988.24 ± 4.92 a	1048.47 ± 2.12 c	1173.74 ± 96.77 d	141.62 ± 8.94 h	255.67 ± 2.30 g	279.93 ± 15.23 f	154.02 ± 0.34 e
	BP	609.19 ± 4.10 c	659.99 ± 8.22 b	1197.04 ± 39.47 a	536.07 ± 0.43 d	97.78 ± 2.79 f	128.96 ± 1.05 e	147.69 ± 4.05 e	95.85 ± 0.44 f
	TP	1780.84 ± 10.65 c	2462.11 ± 46.87 a	2472.77 ± 40.82 a	2015.00 ± 85.27 b	269.75 ± 5.36 e	392.99 ± 1.21 d	450.36 ± 19.26 d	286.28 ± 0.17 e
Isoquercitrin (IQUE)	FP	128.43 ± 7.33 b	216.31 ± 5.46 a	221.65 ± 0.15 a	222.77 ± 5.93 a	10.90 ± 0.40 e	21.06 ± 0.91 d	22.43 ± 0.10 d	40.41 ± 0.02 c
	CP	1172.76 ± 8.93 c	1295.64 ± 6.52 b	1395.58 ± 17.67 a	1363.47 ± 49.30 a	179.10 ± 11.78 e	329.47 ± 3.03 d	361.52 ± 20.15 d	195.58 ± 0.43 e
	BP	192.00 ± 1.07 d	264.96 ± 2.82 b	286.55 ± 9.23 a	212.90 ± 0.76 c	ND	ND	25.20 ± 2.13 f	37.51 ± 0.02 e
	TP	1493.18 ± 0.52 c	1776.91 ± 14.80 b	1903.78 ± 8.29 a	1799.13 ± 56.00 b	190.00 ± 11.38 g	350.53 ± 2.13 e	409.16 ± 22.39 d	273.50 ± 0.43 f
Quercitrin (QUER)	FP	4510.19 ± 286.30 d	20775.72 ± 107.48 a	15001.01 ± 193.94 c	16552.39 ± 371.56 b	518.29 ± 15.12 g	896.19 ± 30.23 e	826.66 ± 6.05 f	887.27 ± 5.59 e
	CP	2558.98 ± 30.23 a	578.75 ± 15.12 d	790.38 ± 15.11 b	639.22 ± 15.30 c	ND	ND	ND	ND
	BP	66900.24 ± 1247.09 c	124417.57 ± 1776.16 a	90709.88 ± 2116.28 b	21936.13 ± 744.58 d	3463.24 ± 121.53 g	3577.77 ± 12.85 g	4419.79 ± 86.46 f	8331.27 ± 48.37 e
	TP	73969.41 ± 1503.16 c	145772.05 ± 1868.52 a	106501.28 ± 1937.45 b	39127.74 ± 357.91 d	3981.52 ± 106.42 h	4473.96 ± 17.38 g	5246.45 ± 80.42 f	9218.55 ± 53.97 e
Populnin (POP)	FP	2.20 ± 0.09 b	5.57 ± 0.91 a	1.81 ± 0.09 b	ND	ND	ND	ND	ND
	CP	34.20 ± 0.25 a	16.06 ± 0.61 c	16.96 ± 0.09 bc	17.86 ± 1.00 b	3.62 ± 0.24 d	ND	1.74 ± 0.18 f	2.93 ± 0.09 e
	BP	ND	ND	ND	ND	ND	ND	ND	ND
	TP	36.40 ± 0.16 a	21.63 ± 0.30 b	18.77 ± 0.01 c	17.86 ± 1.00 c	3.62 ± 0.24 d	ND	1.74 ± 0.18 f	2.93 ± 0.09 e
o-Coumaric acid (o-CC)	FP	8570.31 ± 276.71 d	21464.95 ± 102.74 a	15214.21 ± 194.52 b	14089.54 ± 267.13 c	ND	6493.58 ± 268.50 e	5533.30 ± 6.85 f	5999.06 ± 6.85 ef
	CP	8041.54 ± 210.96 c	12621.03 ± 116.44 a	12515.55 ± 63.01 a	10345.67 ± 142.47 b	5875.77 ± 327.40 d	ND	5845.63 ± 321.92 d	7716.88 ± 28.77 c
	BP	ND	ND	ND	ND	ND	ND	ND	ND
	TP	16611.86 ± 487.68 d	34085.98 ± 13.70 a	27729.76 ± 257.53 b	24435.21 ± 409.59 c	5875.77 ± 327.40 g	6493.58 ± 268.50 g	11378.93 ± 315.07 f	13715.94 ± 35.62 e
Myricetin (MYR)	FP	12524.73 ± 137.64 c	2323.11 ± 204.17 f	17451.74 ± 16.67 a	16729.17 ± 463.96 b	619.87 ± 24.67 h	4092.03 ± 34.20 e	681.29 ± 2.72 g	6524.66 ± 13.78 d
	CP	ND	ND	ND	ND	ND	ND	ND	ND
	BP	2185.55 ± 66.35 b	ND	2499.72 ± 24.31 a	ND	ND	ND	ND	ND
	TP	14710.28 ± 71.29 c	2323.11 ± 204.17 f	19951.46 ± 7.63 a	16729.17 ± 463.96 b	619.87 ± 24.67 h	4092.03 ± 34.20 e	681.29 ± 2.72 g	6524.66 ± 13.78 d
Quercetin (QUE)	FP	12.93 ± 0.10 a	7.85 ± 0.69 c	10.94 ± 0.22 b	3.89 ± 0.07 d	ND	ND	ND	ND
	CP	3.31 ± 0.05 c	2.15 ± 0.04 c	18.73 ± 1.95 a	8.89 ± 0.73 b	ND	ND	ND	ND
	BP	16.36 ± 0.52 b	6.58 ± 0.12 d	35.03 ± 0.88 a	10.86 ± 0.01 c	ND	ND	ND	ND
	TP	32.60 ± 0.37 b	16.58 ± 0.61 d	64.70 ± 2.61 a	23.64 ± 0.81 c	ND	ND	ND	ND
Kaempferol (KAE)	FP	5.59 ± 0.17 c	34.93 ± 0.71 a	2.04 ± 0.15 d	26.17 ± 0.51 b	ND	ND	ND	ND
	CP	ND	2.85 ± 0.18	ND	ND	ND	ND	ND	ND
	BP	ND	ND	4.26 ± 0.09	ND	ND	ND	ND	ND
	TP	5.59 ± 0.17 c	37.78 ± 0.89 a	6.30 ± 0.05 c	26.17 ± 0.51 b	ND	ND	ND	ND

Pe1, Pe2, Pe3, and Pe4 are the peel of W1, W2, W3, and W4, respectively; Pu1, Pu2, Pu3, and Pu4 are the pulp of W1, W2, W3, and W4, respectively. FP, CP, BP, and TP are free phenolic, conjugated phenolic, bound phenolic, and total phenolic, respectively. ND means not detected. Different letters (a–h) indicate there are statistically significant differences among different samples at the same status (*p* < 0.05).

## Data Availability

Data are contained within the article.

## Supplementary Materials

The following supporting information can be downloaded at: https://www.mdpi.com/article/10.3390/antiox11091831/s1, Figure S1: The total ionic chromatograms of free, conjugate, and bound phenolic fractions of wampee peel and pulp by UHPL-Q-Exactive HF-X.

## Abbreviations

W	wampee
Pe	peel
Pu	pulp
DW	dry weight
FP	free phenolic
CP	conjugated phenolic
BP	bound phenolic
FF	free flavonoid
CF	conjugated flavonoid
BF	bound flavonoid
TPC	total phenolic content
TFC	total flavonoid content
GAE	gallic acid equivalents
RE	rutin equivalents
TE	Trolox equivalents
