# Supplementary material for: Free, Conjugated, and Bound Phenolics in Peel and Pulp from Four Wampee Varieties: Relationship between Phenolic Composition and Bio-Activities by Multivariate Analysis
Source: Antioxidants (Basel). 2022 Sep 16;11(9):1831. doi: 10.3390/antiox11091831 (PMC9495965; doi:10.3390/antiox11091831)
Supplement: Supplementary file 1 [file antioxidants-11-01831-s001.zip › antioxidants-1851595-supplementary.pdf]

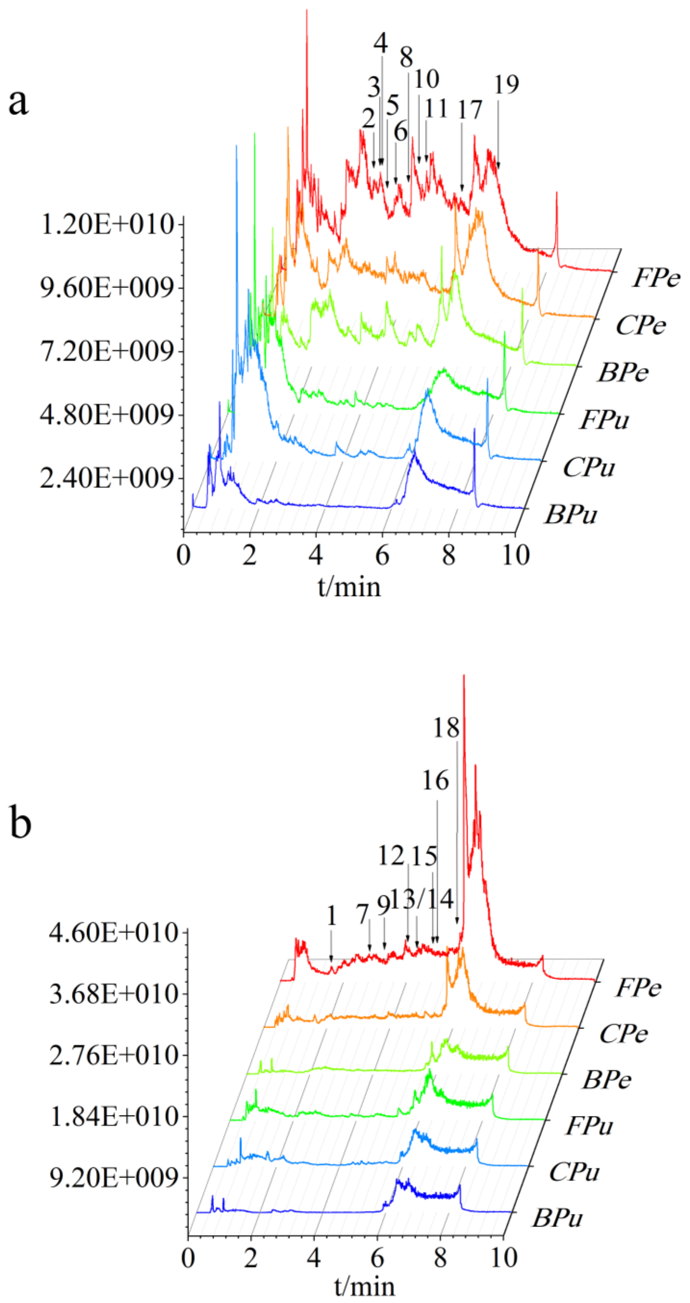

Figure S1. Total ion chromatogram in positive (a) and negative (b) ion mode of free, conjugated, and bound phenolic fractions in the peel and pulp of different wampee samples from South China. FPe/FPu, free phenolic fraction of wampee peel/pulp; CPe/CPu, conjugated phenolic fraction of wampee peel/pulp; BPe/BPu, bound phenolic fraction of wampee peel/pulp.
